# Darier and Ferrand dermatofibrosarcoma of the breast

**DOI:** 10.11604/pamj.2018.31.179.14998

**Published:** 2018-11-14

**Authors:** Nora Naqos

**Affiliations:** 1 Department of Medical Oncology, Mohammed VI University Hospital, Marrakech, Morocco

**Keywords:** Sarcoma, fibrosarcoma, breast

## Image in medicine

Darier and Ferrand dermatofibrosarcoma (DFS) is a cutaneous mesenchymal tumor, rare, representing 0.1% of malignant skin tumors and less than 5% of adult soft tissue sarcomas. The favorite sites are the trunk, followed by the extremities proximal then the head and neck. It often occurs in patients in their 3^rd^-4^th^ decades and presents clinically as plate or nodule lesion. It is a tumor with intermediate malignancy potential, good prognosis after complete resection, slow growth, very high risk of local recurrence, but with low metastatic potential. Due to its rarity, very few studies have been devoted to it. We report an exceptional localization revealed by a mass in the breast. It is a 43-year-old patient who has consulted for an increase in the volume of the right breast, the clinical examination finds a mass ulcero-budding of 12cm/10cm. The biopsy shows the presence of a histiocytofibroma, a mastectomy was performed, and the histopathological examination with immunohistochemistry showed an intense and diffuse CD34 positivity, a focal positivity of AML (smooth muscle actin) and a constant negativity of desmin and PS100 thus confirming that it is a DFS. CT scan does not find any metastasis and a surveillance was adopted. It is a rare tumor and our observation is among of the first cases of mammary siege described in the literature. The main treatment remains a large surgery with margins of resection of at least 4 to 5cm, postoperative radiotherapy is advocated by second recurrence. Systemic chemotherapy is not recommended, rigorous clinical practice must be maintained, because of slow evolution and high recurrence of this tumor.

**Figure 1:**
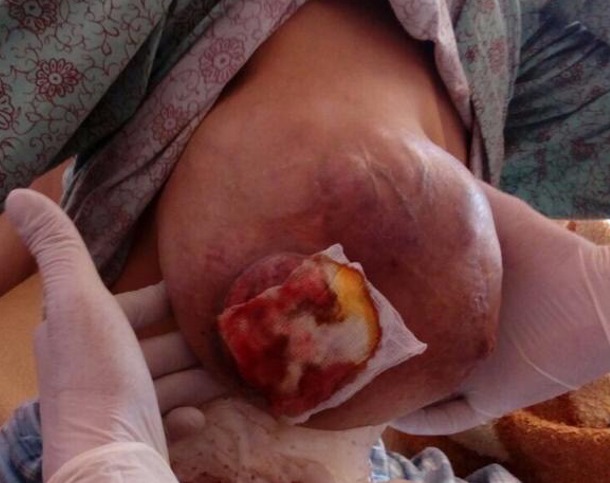
Dermatofibrosarcoma of Darier and Ferrand presented as a mass in the right breast

